# Overproduction and easy recovery of target gene products from cyanobacteria, photosynthesizing microorganisms

**DOI:** 10.1007/s00253-012-3989-0

**Published:** 2012-03-31

**Authors:** Munehiko Asayama

**Affiliations:** Laboratory of Molecular Genetics, College of Agriculture, Ibaraki University, Ami, Inashiki, Ibaraki 300-0393 Japan

**Keywords:** AU-box sequence, Auto cell lysis cyanobacteria, Conjugation, Expression vector, Light-responsive *psbA* promoter

## Abstract

New cyanobacterial expression vectors, possessing an origin of replication that functions in a broad range of Gram-negative bacteria, were constructed. To inspect the shuttle vectors, the gene *gfp* was cloned downstream from the expression control element (ECE) originating from the regulatory region of the *Microcystis aeruginosa* gene *psbA2* (for photosystem II D1 protein), and the vectors were introduced into three kinds of cyanobacteria (*Synechocystis* sp. PCC 6803, *Synechococcus elongatus* PCC 7942, and *Limnothrix*/*Pseudanabaena* sp. ABRG5-3) by conjugation. Multiple copy numbers of the expression vectors (in the range of 14–25 copies per cell) and a high expression of green fluorescent protein (GFP) at the RNA/protein level were observed in the cyanobacterial transconjugants. Importantly, GFP was observed in a supernatant from the autolysed transconjugants of ABRG5-3 and easily collected from the supernatant without centrifugation and/or further cell lysis. These results indicate the vectors together with the recombinant cells to be useful for overproducing and recovering target gene products from cyanobacteria.

**Electronic supplementary material:**

The online version of this article (doi:10.1007/s00253-012-3989-0) contains supplementary material, which is available to authorized users.

## Introduction

Cyanobacteria are photosynthesizing eubacteria that make good models for basic research on photosynthesis and applied biotechnology for the production of carbohydrates, photosynthetic pigments, and other natural products. *Microcystis aeruginosa* strain K-81 (Shirai et al. 1989) and *Synechocystis* sp. strain PCC 6803 are Gram-negative non-nitrogen-fixing cyanobacteria and can perform oxygenic photosynthesis. Light-responsive transcripts of the gene *psbA2* (for photosystem II D1 protein) or their molecular structures have been characterized in unicellular cells of K-81 and PCC 6803 (Asayama et al. 2002; Imamura et al. 2003; Imamura and Asayama 2009; Sato et al. 1996). Moreover, light-responsive K-81 *psbA2* transcripts have also been well studied in heterologous cells of bacillary *Synechococcus elongatus* PCC 7942 (Ito et al. 2003; Shibato et al. 2002).

Gene cloning and transfer are indispensable for cyanobacterial genetic manipulations. In cyanobacteria, possible transformation methods include natural transfer, the electroporation of extraneous DNA, and the transfer of foreign DNA by bacterial conjugation (Golden et al. 1987; Heidorn et al. 2011; Kuhlemeier and van Arkel 1987; Marraccini et al. 1993; Simon 1984; Thiel 1994; Thiel and Wolk 1987). The efficiency of the transformation depends on the donor DNA form and on the competency of the cyanobacterial recipient cells and, as well as in conjugation, on the restriction–modification barriers of the cells (Takahashi et al. 1996). Furthermore, the transferred DNA is classified by fate. One approach is the integration of foreign DNA into the cyanobacterial chromosome. In this case, autonomously replicating plasmids of cyanobacteria cannot be employed, and linearized or circular DNA is generally used during natural transfer and electroporation for the integration into a chromosomal target site via double or single crossover reactions, respectively (Chauvat et al. 1986; Xu et al. 2011). An alternative is the use of a shuttle vector (as *Escherichia coli*–cyanobacteria) which can autonomously replicate in the cytoplasmic space of cyanobacterial cells. In this case, mobilization of the plasmid DNA into the cells is generally performed by conjugation. Since DNA uptake in strain PCC 6803 has been known to be associated with the conversion of double-stranded molecules into single-stranded ones and a low efficiency/high rate of mutagenesis during transformation by electroporation (Barten and Hill 1995), conjugative transfer might be a convenient method in the cyanobacterium.

Vectors mobilized by conjugation into cyanobacteria are categorized by their replicons into two kinds. The first type contains the replication origin of the ColE1-type pMB1 plasmid of narrow *E*. *coli* host range (Thiel 1994); the transfer of plasmids of this type into cyanobacteria is ensured by conjugative plasmids of the IncP group in the presence of the ColK plasmid (Wolk et al. 1984). The second type was constructed on the basis of the replicon from the RSF1010 plasmid belonging to the IncQ group. Plasmids of this kind are able to replicate in a broad range of Gram-negative bacteria, including cyanobacteria and *E. coli* (Kreps et al. 1990). Because IncQ plasmids are of medium size (∼10 kb) with a large replicon, have multiple copy numbers (∼10 to 30 per cell; Huang et al. 2010; Marraccini et al. 1993; Ng et al. 2000; Rawlings and Tietze 2001), and are easy to mobilize (Barth and Grinter 1974; Guerry et al. 1974; Meyer et al. 1982), their use in genetic studies of cyanobacteria seems to be favorable.

Attention has been given to the development and evaluation of expression vectors which can replicate and maintain multiple copies (Elhai et al. 1997; Huang et al. 2010). Here, new cyanobacterial expression vectors were constructed with the expression control element (ECE) of the *M. aeruginosa* K-81 *psbA2* regulatory region and the origin of replication from IncQ plasmid pVZ321, an RSF1010-based shuttle vector (Zinchenko et al. 1999). In this study, a gene encoding green fluorescent protein (GFP) was cloned downstream from the ECE and introduced into the unicellular, bacillary, and filamentous cells of *Synechocystis* sp. PCC 6803, *S. elongatus* PCC 7942, and *Lymnothrix*/*Pseudanabaena* sp. ABRG5-3, respectively. Systematic analyses revealed multiple copy numbers of the vectors in the cells and abundant expression of GFP at the transcriptional and translational levels. Based on the results, the overproduction using autolysed cyanobacteria of target gene products useful to the biotech industry is discussed.

## Materials and methods

### Strains, media, and plasmids

The bacterial strains and plasmids used in this study are listed in Table 1. *Synechocystis* sp. PCC 6803 (Kazusa DNA Research Institute), *S. elongatus* PCC7942, or *Lymnothrix*/*Pseudanabaena* sp. ABRG5-3 cells were grown on CB or BG11 plates or in liquid medium (Allen 1968; Rippka 1988; Shirai et al. 1989) at 30 °C, supplemented with 2.5 or 8 μg ml^−1^ of chloramphenicol as required. All routine plasmid constructions and cloning in *E*. *coli* were done as described (Sambrook and Russell 2000).

**Table 1:** Bacterial strains and plasmids used in this study

Strains or plasmids	Genotype, phenotype and other characteristics	Source or reference
Strains		
*Escherichia coli*	F-endA1 *supE44* λ-*thi*-1 *recA1 gyrA96*	Cosmo Bio
DH5αMCR	*deoR*Δ(*lacZYA-argF*)U169	(Tokyo, Japan)
*Escherichia coli*	*E. coli* B *F* ^*−*^ *ompT hsdS* (r_B_^−^ m_B_^−^) *dcm* ^+^	Stratagene
BL21-CodonPlus	*gal* λ (DE3) *endA* The [*argU ileY leuW* Cm^R^], Tet^R^	(North Torrey Pines RoadLa Jolla, USA)
(DE3)-RIL		
*Synechocystis* sp.	Wild type (WT)	Kazusa DNA
PCC 6803		Research Institute
6803_pVZ321^a^	PCC6803 transconjugant with pVZ321 (Cm^R^, Km^R^)	This study
6803_GFP500	PCC6803 transconjugant with pGFP500 (Cm^R^)	This study
6803_GFP461c	PCC6803 transconjugant with pGFP461c (Cm^R^)	This study
*Synechococcus elongatus*	Wild type (WT)	Univ. of Tokyo
PCC 7942		
7942_pVZ321	PCC7942 transconjugant with pVZ321 (Cm^R^, Km^R^)	This study
7942_GFP500	PCC7942 transconjugant with pGFP500 (Cm^R^)	This study
7942_GFP461c	PCC7942 transconjugant with pGFP461c (Cm^R^)	This study
*Limnothrix*/*Pseudanabaena* sp.	Wild type (WT)	Nishizawa et al. (2010)
ABRG5-3L1		
5-3_pVZ321	ABRG5-3 transconjugant with pVZ321 (Cm^R^, Km^R^)	This study
5-3_GFP500	ABRG5-3 transconjugant with pGFP500 (Cm^R^)	This study
5-3_GFP461c	ABRG5-3 transconjugant with pGFP461c (Cm^R^)	This study
Plasmids		
pAG500	pAM990 + *M. aeruginosa* K-81 *psbA2* ^a^	Agrawal et al. (1999)
	-404/+113 (*Sma*I–*Bgl*II fragment, WT), Ap^R^ Sp^R^	
pAG461	pAM990 + *M. aeruginosa* K-81 *psbA2*	Agrawal et al. (2001)
	−404/+113 (*Sma*I–*Bgl*II fragment, ΔAT), Ap^R^ Sp^R^	
pAD461c	pAM990 + *M. aeruginosa* K-81 *psbA2*	This study
	-404/+113 (*Sma*I–*Bgl*II fragment, ΔAT and a deletion of G at the position of +36), Ap^R^ Sp^R^	
pGLO	*E. coli* expression vector for *gfp*, Ap^R^	Clontech (Mountain View, USA)
R751	IncP Tra^+^, Tp^R^	Meyer and Shapiro (1980)
pVZ321	IncQ Mob^+^, Km^R^ Cm^R^	Zinchenko et al. (1999)
pAM500	Cyanobacterial expression vector (pVZ321 + P*psbA2*_WT), Cm^R^	This study
pAM461c	Cyanobacterial expression vector (pVZ321 + P*psbA2*_ΔAT), Cm^R^	This study
pGFP500	pAM500 + *gfp* derived from pGLO, Cm^R^	This study
pGFP461c	pAM461c + *gfp* derived from pGLO, Cm^R^	This study

*Ap* ampicillin, *Cm* chloramphenicol, *Km* kanamycin, *Sp* spectinomycin, *Tp* trimethoprim

^a^Nucleotide sequences of the pVZ321 vector and *psbA2* of *M. aeruginosa* K-81 were obtained from DDBJ, EMBL, or GenBank under accession numbers AF100175 and D84228, respectively

### Conjugative transformation

Conjugation was done with the host cyanobacteria. *E*. *coli* strain DH5αMCR (Sambrook and Russell 2000) harbored the expression vector, and *E*. *coli* strain JM109 (Sambrook and Russell 2000) harbored the helper plasmid R751, as described previously (Zinchenko et al. 1999). The cyanobacterial transconjugants were selected on CB or BG11 plates supplemented with chloramphenicol (8 μg ml^−1^ for PCC 6803 and ABRG5-3, 2.5 μg ml^−1^ for PCC 7942) or kanamycin (15 μg ml^−1^) under conditions excluding the *E*. *coli* cells.

### DNA, RNA, and protein techniques

Genomic DNA and total RNA were prepared from cyanobacteria as described previously (Imamura et al. 2003). Gel electrophoreses, hybridization, and detection using ECL (enhanced chemiluminescence) systems were reported elsewhere for Southern and Western blots (Imamura et al. 2003). Primer extension was performed as described previously (Asayama et al. 2004) with a specific primer, gloGFP-R (5′-GAATTGGGACAACTCCAGTG-3′). For protein recovery (Fig. 11), the naturally precipitated cell pellet of ABRG5-3 (100 μl) or the supernatant (10 ml) was collected from a screw-cap tube and poured into 50 ml of the cell culture, in which ABRG5-3 transconjugants harboring pVZ321 (Vec) or pGFP461c (−AU) were autolysed. The supernatant was mixed with an equal volume of saturated ammonium sulfate and subjected to centrifugation (13,500 rpm, 10 min) for the precipitation of GFP. The pellet containing GFP was dissolved with 50 μl of TE_10-1_ buffer (Sambrook and Russell 2000). Both fractions from the cell pellet (Cell, 100 μl) or the supernatant (Sup, 50 μl) were dissolved in an equal volume of ×2 SDS sample buffer and subjected to by heat treatment (95 °C, 3 min). Aliquots of respective protein fractions (25 μl) from the cell pellet or supernatant were then subjected to 12.5 % SDS-PAGE. The purification of control GFP from *E. coli* BL21 (Sambrook and Russell 2000) harboring pGLO (Clontech, Mountain View, USA) was carried out by a procedure with hydrophobic interaction chromatography (Bio-Rad, Tokyo, Japan). The GFP polyclonal antibody (rabbit antiserum) was purchased from MBL (Nagoya, Japan). The signal intensity corresponding to the target DNA, RNA (transcripts), or protein on X-ray film was measured with BIO-1D V. 96 software (Vilber Lourmat, Marne la Vallée, France) or a FLA7000 phosphoimager (GE Healthcare, Tokyo, Japan; Asayama et al. 2004).

### Measuring numbers of cells, plasmids, and proteins

The method used to count the cyanobacterial cells was described previously (Asayama et al. 2004; Shirai et al. 1989). For plasmids, the copy number was calculated by comparing the intensity of bands (at position 8.7 kbp) on original X-ray films referring to the samples and concentration markers in the Southern blot analysis (Fig. 2b). Values were expressed as a quota per cell number. For the calculation, we used the value for 70 % efficiency of cell disruption in hot phenol extraction to prepare total DNA (Asayama et al. 2004; Imamura et al. 2003). For proteins (Fig. 4b), the molecule number was also measured by comparing signal bands on original X-ray films referring to the samples and concentration markers (Asayama et al. 2004; Imamura et al. 2003). This value was also expressed as a quota per cell number. The value for 70 % efficiency of cell disruption by the glass–bead method employed to prepare total protein was used (Asayama et al. 2004; Imamura et al. 2003). Of note is that the intensity of signals was standardized using a FLA7000 phosphoimager.

### Polymerase chain reactions

For the analysis of transcripts, reverse transcriptase polymerase chain reaction/quantitative real-time PCR (RT-PCR/QRT-PCR) was done. Details for the preparation of total RNA and cDNA and the RT-PCR/QRT-PCR have been described previously (Asayama et al. 2004). The specific *gfp* primers used were: GFP-F primer, 5′-CATATGGCTAGCAAAGGAGAAGAA-3′ (24 mer); GFP-RT primer, 5′-TTTGTAGAGCTCATCCATGCCATG-3′ (24 mer); and GFP-QRT primer, 5′-GAGAAAGTAGTGACAAGTGTTG-3′). For the measurement of plasmid copy numbers, quantitative PCR (Q-PCR) was also performed. Q-PCR was basically done the same as QRT-PCR with total DNA, prepared from the transconjugants harboring expression vectors and a set of specific primers, 6803rpoB-QF (5′-ATGACAAACCTTGCCACCACGATG-3′, 24 mer) and 6803rpoB-QR (5′-AGCATCCCGTCGCTTGGATTCATC-3′, 24 mer), for the gene *rpoB* (for the β-subunit of RNA polymerase) in the PCC 6803 genomic DNA or primers GFP-F and GFP-RT for the expression vectors.

### Microscopic observation

The cyanobacterial cells grown in the CB or BG11 medium at 30 °C for 12 days were observed under an optical microscope (BX51/DP50, Olympus, Tokyo, Japan). The fluorescence of chlorophyll/phycocyanin (red) or GFP-expressing (green) cells was detected using a U-MWIB2 filter for excitation (460–490 nm) and emission (510 nm; Nishizawa et al. 2010).

## Results

### Construction of cyanobacterial expression vectors carrying an ECE from the *M*. *aeruginosa* gene *psbA2*

**Fig. 1:**
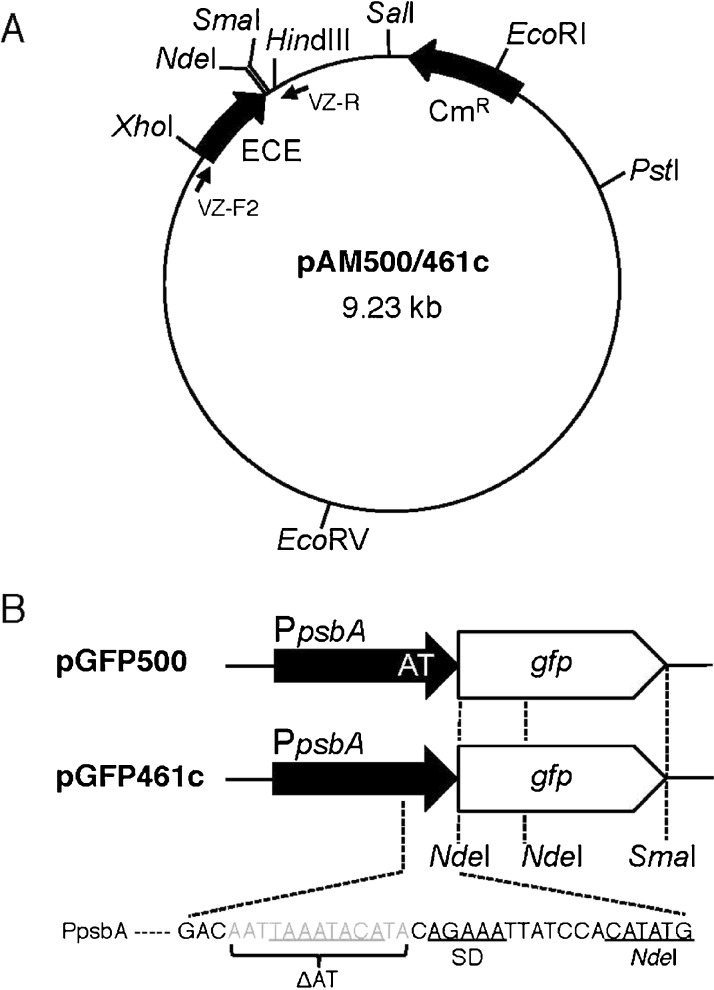
Expression vectors. **a** General structure. The plasmids contain as a regulatory region an expression control element (*ECE*) derived from the promoter of *psbA2* (P*psbA*) of the cyanobacterium *M. aeruginosa* K-81. The set of primers, VZ-F2 and VZ-R, for PCR is shown. **b** Vector for GFP expression. The GFP gene was introduced into the cloning sites of *Nde*I and *Sma*I to create pGFP500 (+AU) and pGFP461c (−AU). The AU-box nucleotide sequence (UAAAUACA) was deleted in pGFP461c for stable accumulation of the GFP mRNA. The nucleotides ATG at the *Nde*I site represent a start codon

The promoter of *psbA2* (P*psbA*) and its ribosome-binding site of the cyanobacterium *M. aeruginosa* K-81 (Agrawal et al. 2001; Horie et al. 2007; Sato et al. 1996) were amplified by PCR using the primers K81psbA-404F_XhoI (5′-CCG*CTCGAG*GATCTCATAGAAACGATAAATC-3′, the site for *Xho*I shown in italics) and HY908-R_NdClSm&Hd (5′-CCC*AAGCTT*TTACTA*CCCGGGATCGTACATATG*TGGATAATTTCTGC-3′, sites for *Hin*dIII, *Sma*I, *Cla*I, and *Nde*I shown in italics) from the plasmid pAG500 (wild-type K-81 *psbA*) or pAD461c (disruption of the AU-box for the light-dependent expression of *psbA2* in the *psbA2* 5′-untranslated region; Agrawal et al. 2001) as the DNA template. The amplified elements were digested with *Xho*I and *Hin*dIII and introduced into the *Xho*I and *Hin*dIII sites of pVZ321, an RSF1010-based shuttle vector (Zinchenko et al. 1999), to make pAM500 or pAM461c (Fig. 1a). The *Nde*I and *Sma*I (or *Hin*dIII) sites were left available for target gene cloning into the pAM vectors. The pAM500 vector contains the light-responsive K-81 *psbA2* promoter and its ribosomal binding site encompassing the AU-box sequence (Fig. 1b) which causes mRNA instability in darkness (Agrawal et al. 2001; Asayama 2006; Horie et al. 2007). In pAM461c, the AU-box sequence was removed to increase mRNA stability, and so the constitutive accumulation of mRNA for a target gene is feasible. In both vectors, the region corresponding to *Xho*I–*Hin*dIII (0.53 kb), which covers most of the kanamycin resistance gene on the original plasmid pVZ321, was consequently replaced with the element of *M. aeruginosa psbA2* (0.5 kb), leaving a chloramphenicol resistance gene as a selective marker gene (Fig. 1a).

### Expression vectors for the overproduction of GFP

Although there are GFPs substituted at certain positions, F64L, S65T, or F64L + S65T (known as eGFP), for the enhancement of fluorescent strength in cells, a normal type of GFP gene was examined in this study (Cormack et al. 1996; Toyoshima et al. 2010; Yoon and Golden 1998). A *Nde*I–*Sma*I fragment of an *Aequorea victoria* GFP gene (717 bp, 239 aa—F64, S65) was prepared as follows. The plasmid DNA of pGLO carrying the GFP gene (from positions 1,343 to 2,059 in the plasmid) was digested with *Sma*I (at 1,080). It was then digested partially with *Nde*I (at 1,340 and 1,574) because one site of *Nde*I is located in the GFP gene (Fig. 1b). A 0.72-kbp fragment of *Nde*I–*Sma*I was isolated and then inserted into the *Nde*I and *Sma*I sites of pAM500 and pAM461c to create pGFP500 and pGFP461c, respectively (Fig. 1b). Nucleotide sequences of the region from *Xho*I to *Hin*dIII on respective plasmids were verified and these vectors were used for bacterial conjugation.

### The GFP transconjugants and plasmid copy number in PCC 6803

**Fig. 2:**
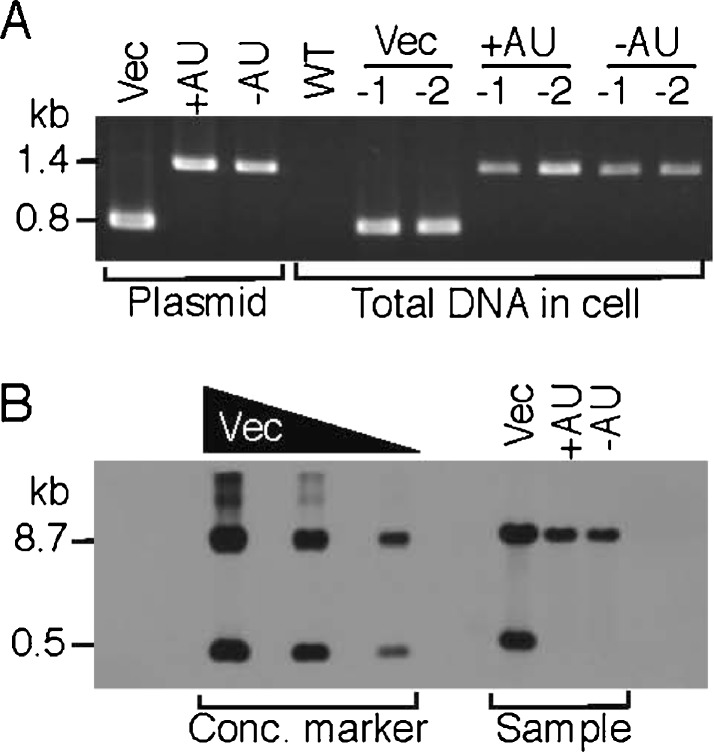
Analysis of PCC 6803 transconjugants containing GFP expression vectors. **a** PCR. Total DNA (1 μg) was isolated from the PCC6803 wild-type (*WT*) or recombinant cells conjugated with pVZ321 (Vec), pGFP500 (+AU), and pGFP4561c (−AU), respectively. Each two samples (−1 and −2) were subjected to PCR with a set of specific primers, VZ-F2 and VZ-R (Fig. 1). The 1.4- or 0.8-kb position for the PCR-amplified fragments from the pGFP500/461c or pVZ321 plasmid DNA is shown as a positive control at the *left*. **b** Southern blot analysis. Total DNA (20 μg) was isolated from the cells in (**a**), digested by the restriction enzymes *Hin*dIII and *Xho*I, and subjected to Southern hybridization with a specific probe (812 bp, a PCR-fragment amplified with pVZ321 and the primers VZ-F2 and VZ-R). The 8.7- or 0.5-kb position for the concentration marker is indicated at the *left*. The DNA of pVZ321 (*left lane*, 0.24 μg, 0.040 pmol; *middle lane*, 0.080 μg, 0.0133 pmol; *right lane*, 0.02 μg, 0.0033 pmol) digested with the same restriction enzymes was subjected to hybridization for the concentration marker which was used for a trial calculation of plasmid copy numbers in the cells (Table 2)

The conjugation with pVZ321 (original vector), pGFP500 (wild-type K-81 *psbA2*, +AU), or pGFP461c (mutagenized K-81 *psbA2*, −AU) was conducted as described previously (see “Materials and methods”). Transconjugants of *Synechocystis* sp. strain PCC 6803 were obtained from the BG11 plates containing chloramphenicol and the presence of the vectors in the cells verified by PCR using the primers VZ-F2 (5′-CTGATGTTACATTGCACAAG-3′) and VZ-R (5′-ATGAAGGAGAAAACTCACCG-3′; Fig. 1a). We confirmed the presence of a 0.8-kb fragment for pVZ321 and a 1.4-kb fragment for pGFP500/461c in the PCR (Fig. 2a). We also verified the copy number of the vectors in the transconjugants (Fig. 2b). When total DNA was prepared from the cells harboring the vectors and Southern analysis was performed with the specific DNA probe (812 bp), which was amplified by PCR using the VZ-F2/-R primers and pVZ321, respective signals at the 0.53-kb (corresponding to a small *Xho*I–*Hin*dIII fragment carrying most of the kanamycin resistance gene in pVZ321) and/or 8.7-kb (corresponding to another large *Xho*I–*Hin*dIII fragment as the vector side) positions were observed on the X-ray film. The copy number of the plasmids was calculated based on the signal intensity at the 8.7-kb position in the Southern blot as follows (also see “Materials and methods”) since the 8.7-kb fragment is common among pVZ321 and its derivatives, pAM and pGFP. For example, the signal intensity from the gel indicated 0.004 pmol of pGFP500 (+AU) or pGFP461c (−AU). This accords with 24 × 10^8^ molecules per lane. On the other hand, the total DNA (7.04 μl/20 μg/lane) prepared from the PCC 6803 transconjugants was subjected to Southern blotting. The volume of 7.04 μl accords with 2.46 ml of the PCC 6803 cell culture (7 × 10^7^ cells per milliliter, Table 2) if the values are based on a 70 % efficiency of cell disruption (“Materials and methods”; Table 2). The culture of 2.46 ml accords with 1.72 × 10^8^ cells per lane. Therefore, the copy number of pGFP plasmids is 14 molecules per cell (=24 × 10^8^ molecules/1.72 × 10^8^ cells). Following the same calculation procedure, the copy number of pVZ321 (Vec) was determined as 18 since the signal intensity from the gel accorded to 0.005 pmol (Table 2). IncQ-type plasmids derived from the RSF1010 replicon have been shown to have copy numbers in *Synechocystis* PCC 6803 ranging from 10 to 30 (or more higher) per cell (Huang et al. 2010; Marraccini et al. 1993; Ng et al. 2000; Rawlings and Tietze 2001). The replicative stability of the RSF1010 replicon depends upon its copy number and was shown to deteriorate when the copy number was lowered (Becker and Meyer 1997; Meyer 2009). Previous reports revealed that the number of PCC 6803 chromosomes was 12 per cell (Labarre et al. 1989). This number was used to create a link to plasmid copy numbers (plasmid number per chromosome number).

**Table 2:** Vector copy numbers and GFP expression levels in cyanobacterial transconjugants

Strain	Vector	Cm^a^ (μg ml^−1^)	Cell no.^b^ (ml^−1^)	Vec. no.^c^ (per cell)	GFP no.^c^ (per cell)	GFP exp. (% per TCP)	Exp. rate ([GFP no.] [Vec. no.]^−1^)
PCC 6803	pVZ321	8	7 × 10^7^	18	N.D.	N.D.	N.D.
	pGFP500	8	7 × 10^7^	14	9.4 × 10^4^	1	6.7 × 10^3^
	pGFP461c	8	7 × 10^7^	14	4.7 × 10^5^	5	3.4 × 10^4^
PCC 7942	pVZ321	2.5	1 × 10^8^	29	N.D.	N.D.	N.D.
	pGFP500	2.5	1 × 10^8^	21	1.9 × 10^4^	0.2	9.0 × 10^2^
	pGFP461c	2.5	1 × 10^8^	22	9.4 × 10^4^	1	4.3 × 10^3^
ABRG5-3	pVZ321	8	8 × 10^7^	16	N.D.	N.D.	N.D.
	pGFP500	8	8 × 10^7^	15	2.0 × 10^4^	0.2	1.3 × 10^3^
	pGFP461c	8	8 × 10^7^	15	1.0 × 10^5^	1	6.7 × 10^3^

*exp*. Expression, *TCP* total cellular protein, *N.D.* not detected

^a^Cm, final concentration of chloramphenicol

^b^Cell no., cell number (colonies from the cell culture were counted on a plate; see text)

^c^Values are based on a 70 % efficiency of cell disruption (see “Materials and methods”)

The copy number of pGFP carrying the *gfp* gene in the PCC 6803 cell was also confirmed by Q-PCR using the total DNA and a set of specific primers for *gfp* or *rpoB*, a single copy gene in the PCC 6803 chromosome (no other *rpoB* gene exists on the six kinds of endogenous plasmids in the cell; genome database, http://genome.kazusa.or.jp/cyanobase). The results indicated that the copy number of pGFP was 25 when the copy number of *rpoB* was 12 per PCC 6803 transconjugant (Electronic supplementary material (ESM) Fig. S1). This result did not contradict that of the Southern analysis mentioned above.

### Analysis of light-responsive target gene expression from the vectors at the RNA level in PCC 6803

**Fig. 3:**
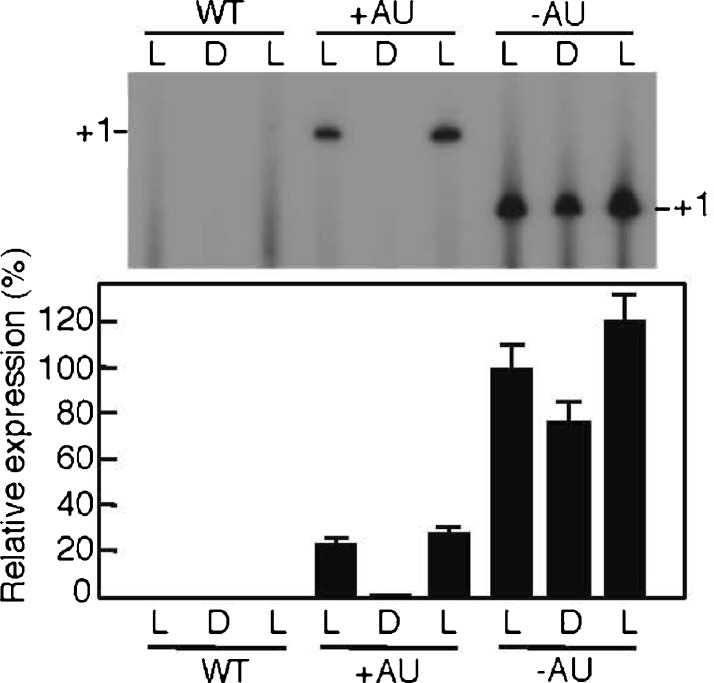
GFP expression at the RNA level in PCC6803. The *Synechocystis* sp. PCC 6803 cells of the wild type (*WT*) or transconjugants harboring pGFP500 (+AU) or pGFP461c (−AU) were grown under continuous white light (35 μE m^-2^ s^−1^) in the CB medium for 12 days, then exposed to a 12-h light (*L*)/12-h dark (*D*)/12-h light (*L*) cycle. The cells were harvested at specific times [3 h (L), 15 h (D), and 27 h (L)]. Total RNA (10 μg) was prepared from the cells and subjected to primer extension analysis with the ^32^P-end-labeled specific primer, gloGFP-R. Relative signal intensities (+1 as the transcription start point) on an X-ray film are presented for transcripts, with a 100 % value referring to that after 3 h of light in the GFP461c strain. Relative average values (in percent) are shown as *bars* with error values (standard deviation, SD) obtained from independent triplicate experiments

The accumulation of GFP transcripts from the expression vectors was examined by primer extension using the GFP-specific primer gloGFP-R. The results are shown in Fig. 3. When total RNA was prepared from the PCC 6803 cells harboring pGFP500 (+AU) and subjected to analysis, a clear pattern of light-responsive transcripts was observed under the light–dark–light (L–D–L) condition (Fig. 3, top; Agrawal et al. 2001; Asayama 2006; Horie et al. 2007). On the other hand, abundant GFP transcripts were observed in the PCC 6803 cells harboring pGFP461c even in darkness (Fig. 3, top). The position corresponding to the 5′-end (+1 as a transcription start point) of the GFP transcript shifted downstream 13 nt in the cells harboring pGFP461c, compared with that in the cells carrying pGFP500, since the AU-box sequence (13 bp in the pGFP461c; Fig. 1b) was deleted from the 5′-untranslated region (5′-UTR) of K-81 *psbA2*. The amount of the GFP transcripts from the cells harboring pGFP461c (−AU) was approximately five times higher than that from the cells harboring pGFP500 (+AU) under light (Fig. 3, bottom). This finding is in agreement with previous reports of the abundant accumulation of the K-81 mutagenized *psbA2* (−AU) transcripts in transformants of *S*. *elongatus* PCC 7942 (Agrawal et al. 2001; Asayama 2006; Horie et al. 2007) and that the ECE is functional in the expression vectors at the RNA level.

### Overexpression of the target gene product at the protein level in PCC 6803

**Fig. 4:**
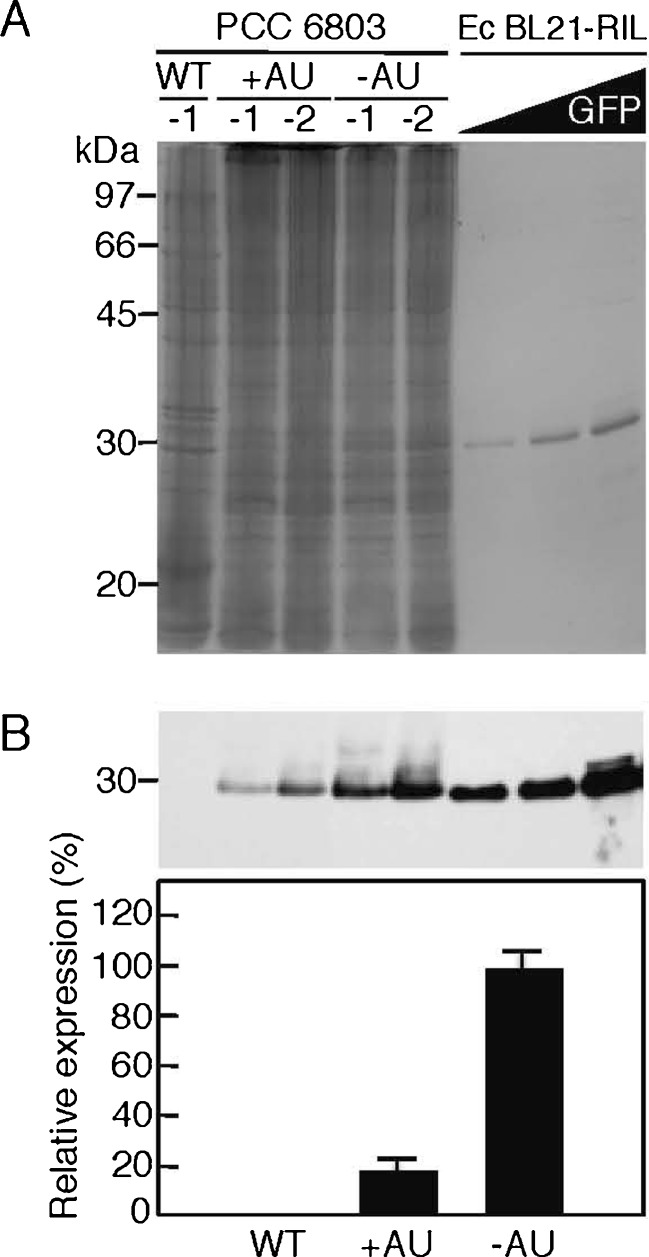
GFP expression at the protein level in PCC6803. **a** SDS-PAGE. Total cellular protein (40 μg) was prepared from wild-type (*WT*) *Synechocystis* sp. PCC6803 cells or cells harboring pGFP500 (+AU) or pGFP461c (−AU) grown in the CB medium for 12 days, then each two samples (−1 and −2) were subjected to 12.5 % SDS-PAGE. The gel was stained with Coomassie Brilliant Blue R-250. Purified GFP (29 kDa) proteins (*left lane*, 1.45 μg, 50 pmol; *middle lane*, 2.90 μg, 100 pmol; *right lane*, 8.70 μg, 300 pmol) obtained from *E. coli* BL21 (RIL as codon plus) harboring pGLO were also applied as concentration markers. The positions (in kilodaltons) from a standard molecular size marker are shown at the *left*. **b** Western blot analysis. *Top* Aliquots of total cellular protein (40 μg) loaded from the samples shown in the different lanes in Fig. 4a in the same order were subjected to Western blotting with a specific antibody for GFP. The 30-kDa position is indicated at the *left*. Signal intensities corresponding to GFP on an X-ray film in the *top panel* were measured and presented as relative values (pVZ461c as 100 %) with error bars (*n* = 3, means ± SD)

GFP expression was inspected at the protein level in the transconjugants (Fig. 4). When two fractions (−1 and −2) of total cellular protein were prepared from the cells and subjected to SDS-PAGE, bands were observed at the 29-kDa position (Fig. 4a). The intensity of the bands accounted for approximately 1 % and 5 % of total protein prepared from the cells harboring pGFP500 and pGFP461c, respectively. We further examined whether the bands were GFP proteins. We observed a signal at 29 kDa in the pGFP500 (+AU)- and pGFP461c (−AU)-harboring cells by Western blotting using the specific GFP polyclonal antibody, whereas there was no signal in the wild-type cells as a negative control, indicating that GFP was expressed in the transconjugants (Fig. 4b). The number of proteins was also calculated based on the signal intensity at the 29-kDa position in the Western blot as follows (also see “Materials and methods”). For example, the signal intensity from the gel indicated 69 pmol of GFP from pGFP461c (−AU)-harboring cells. This accords with 4.14 × 10^13^ molecules per lane. On the other hand, the total protein (20.4 μl/40 μg/lane) prepared from the PCC 6803 transconjugants carrying pGFP461c was subjected to Western blotting. The volume of 20.4 μl accords with 1.26 ml of the PCC 6803 cell culture (7 × 10^7^ cells per milliliter; Table 2) if the values are based on a 70 % efficiency of cell disruption (“Materials and methods”; Table 2). The culture of 1.26 ml accords with 8.79 × 10^7^ cells per lane. Therefore, the number of GFP molecules expressed in the PCC 6803_pGFP461c transconjugant is 4.7 × 10^5^ per cell (=4.14 × 10^13^ molecules/8.79 × 10^7^ cells). Using the same calculation procedure, the number expressed in the PCC 6803_pGFP500 transconjugant was determined as 9.4 × 10^4^ since the signal intensity from the gel accorded with 13.8 pmol (Table 2). This value well coincided with the result in Fig. 3.

### Microscopic observation for GFP overexpressed in PCC 6803 cells

**Fig. 5:**
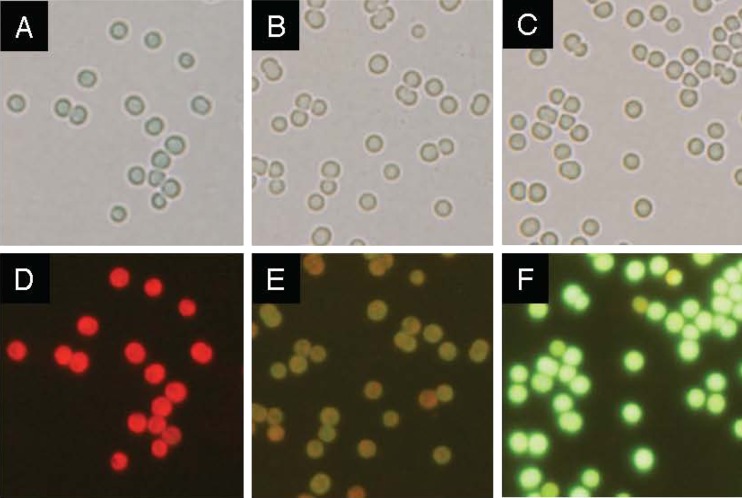
GFP expression in PCC6803. *Synechocystis* sp. PCC 6803 cells were grown in the CB medium for 12 days. Wild-type cells (**a**), or cells harboring pGFP500 (**b**) or pGFP461c (**c**), were subjected to microscopic observation. Fluorescence of GFP was also observed in the wild-type cells (**d**), or cells harboring pGFP500 (**e**) or pGFP461c (**f**). The exposure time of all samples was 0.7 s

Under the optical microscope, the shape and size appeared the same among the wild-type cells (Fig. 5a) and transconjugants (Fig. 5b, c). The transconjugants appeared slightly brown when grown in the CB medium supplemented with chloramphenicol. Fluorescent microscopic observation was conducted for the cells of the transconjugants. We observed browny green (panel e) and green (panel f) cells harboring pGFP500 and pGFP461c, respectively, against the red cells (panel d) of the wild type. This also showed significant GFP expression in transconjugants with the pGFP vectors.

### Stable expression of GFP in PCC 6803

**Fig. 6:**
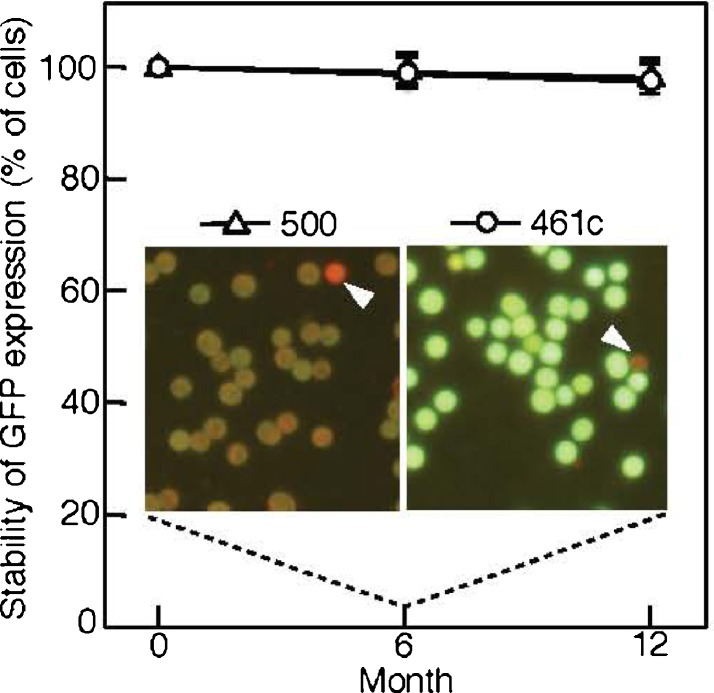
Stability of GFP expression over time in long-term cultures of *Synechocystis* sp. PCC 6803 transconjugants. The *Synechocystis* sp. PCC 6803 transconjugants harboring pGFP500 (*triangles*) or pGFP461c (*circles*) were inoculated into Erlenmeyer flasks (1 %, *v*/*v*, in 50 ml of the CB medium, 110 rpm) at a regular interval of 3 weeks and continuously cultivated with the repetitive inoculation for 12 months. At 3, 6 (*inset*), and 12 months, the cells were harvested and again subjected to microscopic observation for fluorescence of GFP. For evaluation of GFP expression, five windows containing approximately 25–50 cells were randomly selected in the microscopic field, and the percentages for the numbers of green cells (expressed GFP) versus red cells (not expressing GFP, *arrowhead*) are shown as the stability of GFP expression. The *line* (*triangles*, *circles*) *graph* is presented with a 100 % value referring to that of the start of incubation (0 month, see Fig. 5e, f). Relative average values are shown as *bars* with error values (*n* = 5, means ± SD)

To address the stable expression of GFP in PCC 6803 cells, transconjugants were cultivated with repetitive inoculation in which the cells were transferred every 3 weeks to new CB medium (8 μg ml^−1^ of chloramphenicol) in Erlenmeyer flasks for 12 months. The number of cells expressing GFP was almost the same during the period and the ratio (cells not expressing GFP/total transconjugants) very slightly decreased within 2.5–5 % in 6–12 months (Fig. 6). This suggests that the expression vectors had been constantly maintained in the PCC 6803 cells and that expression of GFP apparently occurred. Six and 12 months may correspond respectively to about 400–500 and 800–1,000 generations (Liu et al. 2011; Shen et al. 1993).

### Medium’s effect on GFP expression

**Fig. 7:**
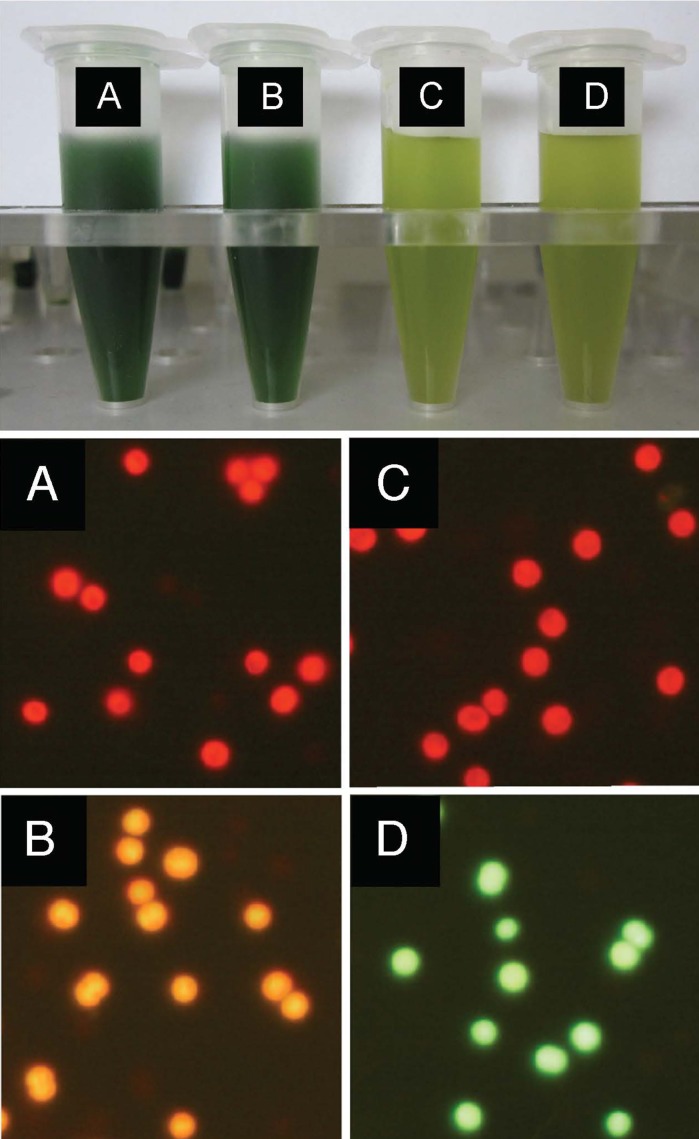
Medium’s effect on GFP expression. *Synechocystis* sp. PCC 6803 cells grown in the BG11 (**a**, **b**) or CB (**c**, **d**) medium for 12 days, harboring pVZ321 (**a**, **c**) or pGFP461c (**b**, **e**), were harvested into microtubes (*top*) and subjected to microscopic observation (*bottom*). Conditions were as for Fig. 5, but the exposure time was 0.5 s

It was examined whether different levels of GFP expression occur in the PCC 6803 transconjugants grown in different media. The results are shown in Fig. 7. The cells harboring pVZ321 (the original vector, −GFP) appeared red when cultivated in both the BG11 (panel a) and CB (panel c) media and expressed no GFP. On the other hand, the cells harboring pGFP461c (+GFP) appeared orange when grown in the BG11 medium (panel b), but green when grown in the CB medium (panel d). The signal intensity corresponding to GFP prepared from the cells shown in panel b was actually lower than that in panel d when equal amounts of total protein were subjected to Western blotting (data not shown). These results show an effect of the medium and that GFP expression is apparently higher in the PCC 6803 cells grown in the CB medium than in the BG11 medium.

### The GFP transconjugants and plasmid copy number in PCC 7942 and ABRG5-3

**Fig. 8:**
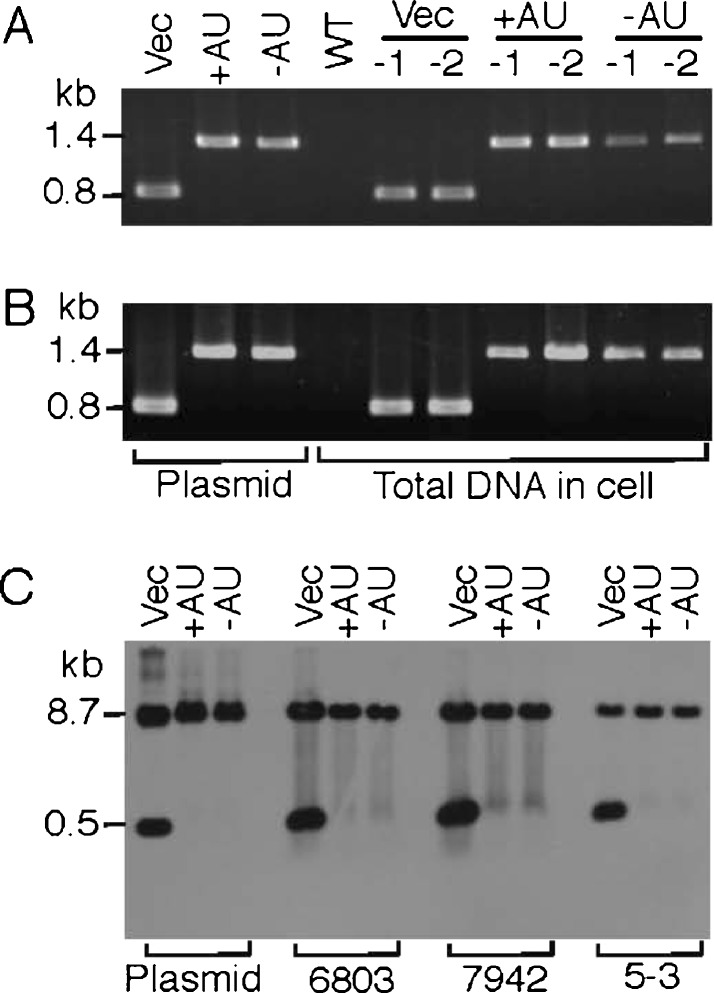
PCR analysis of recombinant *S*. *elongatus* PCC 7942 (**a**) and *Lymnothrix*/*Pseudanabaena* sp. ABRG5-3 (**b**) containing GFP expression vectors. Per strain, 1 μg of total DNA was analyzed by the same procedure as described in Fig. 2a. **c** Southern blot analysis of each expression vector’s copy number in PCC 7942 and ABRG5-3 transconjugants. The DNA of pVZ321 (Vec), pAM500 (+AU), and pAM461c (−AU) (*left block*, 0.0133 pmol) digested with the restriction enzymes *Hin*dIII and *Xho*I was subjected to hybridization with a specific probe as described in Fig. 2b for the concentration marker which was used for the trial calculation of plasmid copy numbers in the cells (Table 2). Total DNA (20 μg) was isolated from the respective transconjugants of PCC 6803, PCC 7942, and ABRG5-3, digested by the restriction enzymes, and also subjected to hybridization with the specific probe. Of note is that the exposure time for the X-ray film was approximately 1.5 times longer than that of Fig. 2b

Since the original pVZ plasmids have a broad host range in cyanobacteria (Zinchenko et al. 1999), it was examined whether the pAM expression vectors can be used in PCC 7942 as a bacillar and ABRG5-3 as a filamentous cyanobacterium (Nishizawa et al. 2010). The results in Fig. 8 show that plasmids pVZ321 (Vec), pGFP500 (+AU), and pGFP461c (−AU) were all maintained in the PCC 7942 and ABRG5-3 cells (panels a and b). It was also confirmed that multiple copy numbers of these expression vectors exist in PCC 7942 (Table 2). Although the expression vectors might also exist in multiple copy numbers in an ABRG5-3 transconjugant, the cell number per milliliter of the cell culture was calculated based on the number of colonies that appeared on CB medium after dilution of the cell culture. Since ABRG5-3 is a filamentous multicellular cyanobacterium, it was difficult to acquire an accurate cell number per milliliter of the cell culture (Nishizawa et al. 2010). Therefore, the estimated cell number described above was used for the calculation of plasmid copy numbers in the ABRG5-3 transconjugants. For example, the signal intensity from the gel indicated 0.002 pmol of pGFP500 (+AU) or pGFP461c (−AU). This accords with 12 × 10^8^ molecules per lane. On the other hand, the total DNA (7.04 μl/20 μg/lane) prepared from the ABRG5-3 transconjugants was subjected to Southern blotting. The volume of 7.04 μl accords with 1 ml of the ABRG5-3 cell culture (8 × 10^7^ cells per milliliter; Table 2) if the values are based on a 70 % efficiency of cell disruption (“Materials and methods”; Table 2). The 1 ml of culture accords with 8 × 10^7^ cells per lane. The copy number of pGFP plasmids may be thus 15 molecules per cell (=12 × 10^8^ molecules/8 × 10^7^ cells). Following the same calculation procedure, the copy number of pVZ321 (Vec) was determined as 16 since the signal intensity from the gel accorded with 0.0021 pmol (Table 2).

### Analysis of the target gene expression at the RNA level in ABRG5-3

**Fig. 9:**
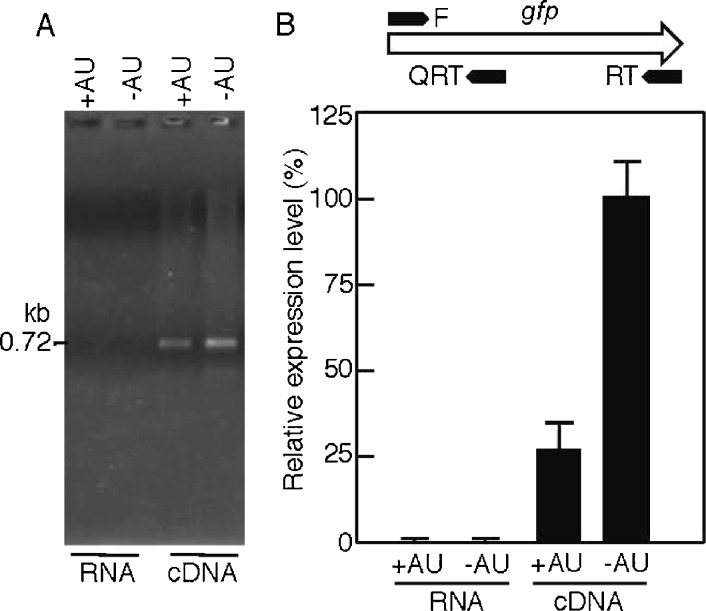
GFP expression at the RNA level in ABRG5-3 transconjugants. **a** RT-PCR. The ABRG5-3 transconjugants harboring pGFP500 (+AU) or pGFP461c (−AU) were grown under continuous white light illumination (35 μE m^−2^ s^−1^) in the BG11 medium for 12 days. The cells were harvested, total RNA (0.1 μg) without genomic DNA was prepared from the cells, and then cDNA (0.1 μg) was synthesized from the template RNA with the GFP-specific primer GFP-RT and reverse transcriptase. The RNA and cDNA were subjected to RT-PCR with the specific primers GFP-F and GFP-RT. The position of 0.72 kb for *gfp* is shown at the *left*. **b** QRT-PCR. The respective RNAs and cDNA were subjected to QRT-PCR with the GFP-specific primers GFP-F and GFP-QRT. The relative positions of each primer within the *gfp* gene are shown at the *top*. Details for the PCR were described previously (Asayama et al. 2004). Relative expression levels for transcripts were calculated with a 100 % value referring to that of cDNA in the GFP461c strain. Relative average values (in percent) are shown as *bars* with error values (standard deviation, SD) obtained from independent triplicate experiments

The accumulation of GFP transcripts in the transconjugants of ABRG5-3 was examined by PCR (Fig. 9). When total RNA was prepared from the cells harboring pGFP500 (+AU) and pGFP461c (−AU) and subjected to RT-PCR, no bands were observed, indicating the RNA template to be of appropriate quality and to contain no genomic DNA (panel a, left). Clear bands at the 0.72-kb position referring to the GFP transcript were observed when the cDNA samples were used in RT-PCR with the primers GFP-F and GFP-RT (panel a, right; panel b, top). This indicates that the GFP transcripts were expressed in the ABRG5-3 transconjugants. To confirm the precise amount of the GFP transcripts, we further conducted a QRT-PCR analysis using the cDNA (panel b). When GFP-F and GFP-QRT (panel b, top) were used, the apparent GFP transcripts were observed, and the amount of the GFP transcripts from the cells harboring pGFP461c (−AU) was approximately four times higher than that from the cells harboring pGFP500 (+AU) under light.

### Microscopic observation for GFP overexpressed in the ABRG5-3 cells

**Fig. 10:**
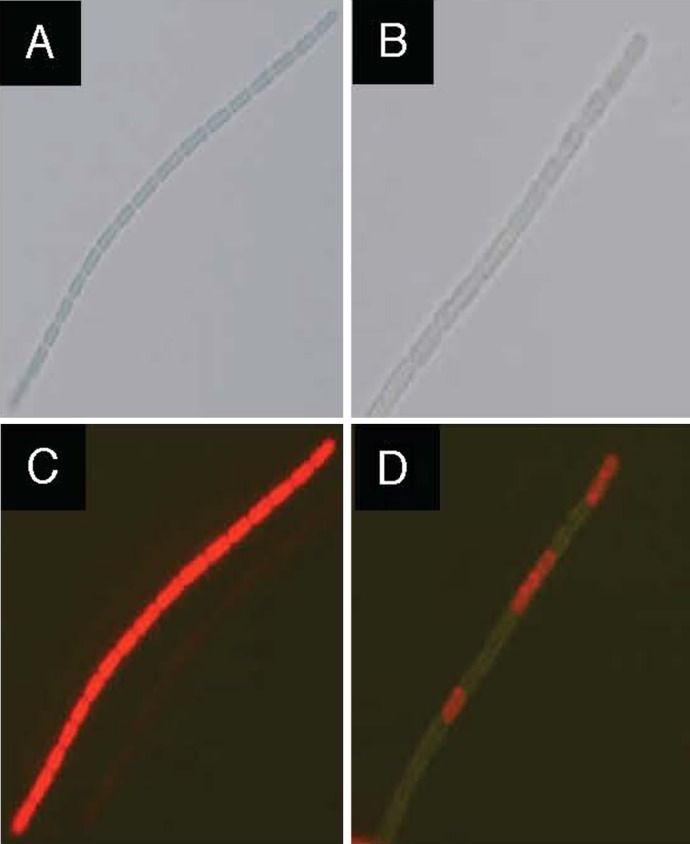
GFP expression in ABRG5-3 transconjugants. ABRG5-3 cells were grown in the CB medium for 12 days. Wild-type cells (**a**) or cells harboring pGFP461c (**b**) were subjected to microscopic observation. Fluorescence was also observed in the wild-type cells as a *red color* (**c**), or pGFP461c-harboring cells as a *dark green/orange color* (**d**) with a fluorescence microscope. Other details were as described for Fig. 5

Fluorescent microscopic observation was conducted in the ABRG5-3 cells (Fig. 10). It was confirmed that browny green (panel d) cells harbored pGFP461c against the glowing red cells (panel c) of the wild type. This also showed apparent GFP expression in the transconjugants harboring pGFP461c. The vivid red color (panel c) is unique to wild-type cells of ABRG5-3 and depends on an abundance of photosynthetic pigments (e.g. phycocyanin), as reported previously (Nishizawa et al. 2010). The abundant accumulation of photosynthic pigments according to the red cell color may hinder observations of the green color of GFP expressed in the transconjugants (panel d).

### Easy recovery of overproduced GFP from autolysed ABRG5-3 cells

**Fig. 11:**
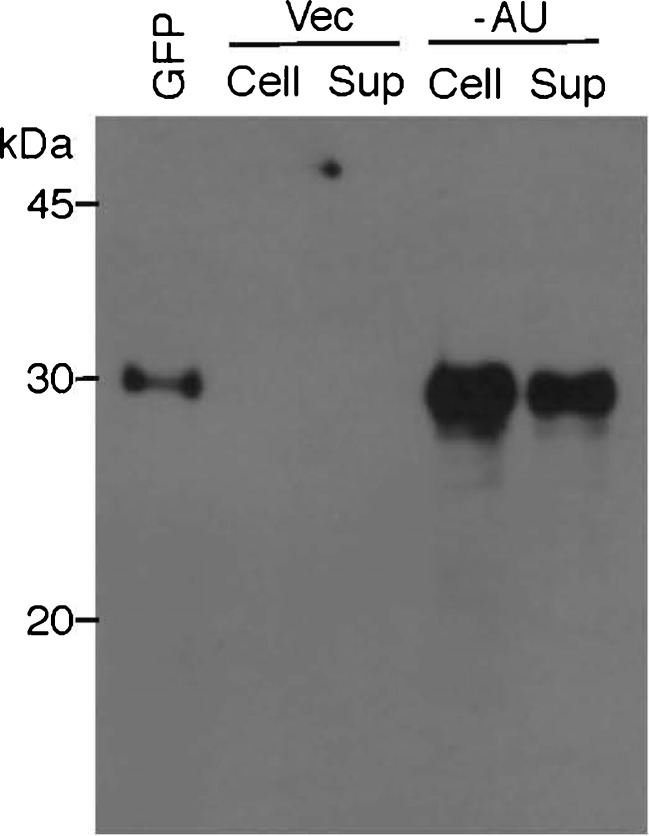
Western blot analysis for confirmation of overexpression and easy recovery of GFP from autolysed ABRG5-3 transconjugant cells. The ABRG5-3 transconjugants harboring pVZ321 (Vec) or pGFP461c (−AU) were grown in the CB medium under continuous white light illumination (100 μE m^-2^ s^−1^) at 30 °C with shaking (50 rpm) in Erlenmeyer flasks (50 ml of medium in a 300-ml flask exposed to 2 % CO_2_ gas) for 7 days. After this phase of cell growth for overproduction of GFP, the cell culture (50 ml) was poured into a screw-cap tube and allowed to stand for 3 days on a laboratory bench at room temperature for promoting auto-cell lysis. Cell sediment (100 μl) corresponding to the 50-ml cell culture or protein precipitate (50 μl) from the supernatant corresponding to 10 ml of the 50-ml culture (see details described in “Materials and methods”) was mixed with an equal volume of ×2 SDS sample buffer, then subjected to 12.5 % SDS-PAGE. The gel was also subjected to Western blot analysis with the GFP antibody as in Fig. 4. Purified GFP (29 kDa) protein (1 μg) was also applied as a size marker (*left lane*)

An attempt was made to recover the target gene product, GFP, from the supernatant of ABRG5-3 cells harboring pGFP461c (Fig. 11). It has been reported that auto-cell lysis occurs gradually (>50 % cells) within several days when ABRG5-3 cells are first subjected to liquid culture for cell growth and the accumulation of products and then exposed to static (standing) cultivation for auto-cell lysis (Nishizawa et al. 2010). In this study, GFP was shown to ooze into the supernatant (60 % of all cells autolysed) from the ABRG5-3 transconjugant harboring pGFP461c in the CB medium. Subsequently, the fractions of GFP (“Materials and methods”) collected from the cell (Cell, total GFP in 40 % of living cells) or the supernatant (Sup, total GFP in 60 % of autolysed cells) were subjected to Western blot analysis. The signal intensity for GFP from the gel accords with 3.2 × 10^12^ molecules per milliliter of the cell culture (as the remaining cell pellet, 40 %) or 4.8 × 10^12^ molecules per milliliter of the cell culture (as released into supernatant, 60 %). The total amount of GFP expressed in 5-3_pGFP461c was thus ${8} \times {1}{0^{{{12}}}}\left[ { = \left( {{3}.{2} + {4}.{8}} \right) \times {1}{0^{{{12}}}}} \right]$ molecules per milliliter of the cell culture. Therefore, the number of GFP molecules expressed in the 5-3_pGFP461c transconjugant is 1.0 × 10^5^ per cell (=8 × 10^12^ molecules/8 × 10^7^ cells). Using the same calculation, the number expressed in the 5-3_pGFP500 transconjugant was determined as 2.0 × 10^4^ molecules per cell (Table 2). The analysis revealed that the supernatant from the autolysed cells harboring pGFP461c contains GFP molecules and that the GFP released was easily collected from the supernatant.

## Discussion

To my best knowledge, this is the first report including simultaneous analyses at the levels of DNA, RNA, and protein for a target gene, cloned into an expression vector, in photosynthesizing bacteria. In this study, the cyanobacterial *psbA2*-type promoter (P*psbA*) and its encompassing region with the AT- (as AU- on RNA) box sequence from *M*. *aeruginosa* K-81 (Agrawal et al. 2001; Asayama 2006; Horie et al. 2007; Sato et al. 1996) were used for the expression control element (ECE) in two expression vectors, pAM500 and pAM461c. The expression of the target gene depends on the light conditions when transconjugants harbor pAM500 carrying the ECE of wild-type *psbA2* (with AU-box, +AU). The usage of pAM500 with the light-controlled ECE seems, however, relatively limited since in many instances it would be more useful to manipulate cyanobacteria able to grow in the dark. Nevertheless, the wild-type P*psbA* could be used for moderate gene expression that should be turned off during the night. This might be suitable for some restricted accumulation of a target gene product which is toxic in cyanobacteria since the overexpression of target proteins sometimes causes growth inhibition and/or results in inclusion body formation in host cells. On the other hand, the use of pAM461c with the ECE lacking the AU-box sequence (−AU) might be useful to overproduce target gene products under light and dark conditions. These two approaches confer an advantage or a variation for overexpression of the target gene in cyanobacteria.

Previous reports revealed that a shuttle vector, pARUB19, based on an endogenous *Synechococcus* plasmid and carrying a RuBisCO promoter, P*rbc*, and an ampicillin resistance marker could confer recombinant luciferase production at a level equivalent to 1.2 % of the total soluble protein in *Synechococcus* sp. PCC 6301 cells (Takeshima et al. 1994). In another report, a IncQ broad-host-range BioBrick shuttle vector, pPMQAK1, was constructed and derivatives with promoters (P*trc*, P*rbc*, P*lac*, P*tet*, P*R*, and P*rnpB*) and a *gfp* gene were tested. Expression from the vectors in *Synechocystis* sp. PCC 6803, however, partially depended on the presence of a *lac* operator system derived from *E*. *coli* (Huang et al. 2010). Moreover, an integrative expression vector, pFPN, placed in the non-coding region of the genome of the filamentous cyanobacterium *Anabaena* sp. PCC 7120 was constructed with the strong light-inducible *Anabaena* promoter P*psbA1* by Chaurasia’s group (Chaurasia et al. 2008). The *psbA1* promoter could drive expression from the subcloned *gfp* gene and GFP was observed in the cells. *Synechocystis* sp. PCC 6803 has also been reported to have regulated promoters: P*petJ* (from the cytochrome c6 gene), which is repressed by copper (Tous et al. 2001), and P*clpP* (from a protease gene) and P*rbpP* (from an RNA-binding protein gene), which are responsible for the circadian regulation of bioluminescence production when inserted in a promoter-trap vector upstream of a luciferase gene (Aoki et al. 2002). The examples and data from this study show that various promoters are at hand for possible use as expression vectors.

The medium-related effects of fluorescence expression in this study were interesting and the use of the CB liquid medium rather than the BG11 liquid medium was more convenient in this study (Fig. 7). The reason for this is still unclear. The BG11 medium is relatively abundant in nitrogen added as sodium nitrate (Allen 1968; Rippka 1988). The nitrogen-rich medium allows the accumulation of photosynthetic pigments in cells, resulting in the brilliant blue-green color of the BG11 culture (Fig. 7, top, panels a and b versus. c and d) which relates to the strong red color as a background in the fluorescent microscopic observation. The CB medium was adjusted to pH 9.0, and thus more alkaline than the BG11 medium (pH 7.6) when the cells were inoculated for cultivation. How these differences in the composition of the culture medium influence microscopic observations and/or P*psbA* function in the transformed cell remain to be elucidated (Mulo et al. 2009).

A filamentous and non-heterocystous cyanobacterium, *Limnothrix*/*Pseudanabaena* ABRG5-3, has been isolated (Nishizawa et al. 2010). This strain allowed transconjugation with pVZ321 and showed an abundant accumulation of pigments, easy recovery of nucleic acids, and autolysis of cells under static cultivation. The results in this study showed easy recovery of the non-secreted target gene product using the auto-cell lysis of ABRG5-3 transformed with an expression vector (Fig. 11). Since auto-cell lysis can be quite convenient for the easy recovery of overproduced materials without complicated cell destruction and centrifugation, we are trying to obtain transconjugants of ABRG5-3 overproducing value-added materials and to recover the products from the autolysed cells as a next step. Because the ability of cyanobacteria to photosynthesize is relatively high, it makes sense that CO_2_ gas is effectively fixed by photosynthesis in cyanobacteria producing high-value products, for example, pigments, carbohydrates, and fuels (Ducat et al. 2011).

## Electronic supplementary material

Below is the link to the electronic supplementary material.

ESM 1 (PDF 52 kb)
