# Supplementary material for: Overproduction and easy recovery of target gene products from cyanobacteria, photosynthesizing microorganisms
Source: Appl Microbiol Biotechnol. 2012 Mar 31;95(3):683–95. doi: 10.1007/s00253-012-3989-0 (PMC3396340; doi:10.1007/s00253-012-3989-0)
Supplement: Supplementary file 1 — (PDF 52 kb) [file 253_2012_3989_MOESM1_ESM.pdf]

**MS title:** Overproduction and easy recovery of target gene products from cyanobacteria, photosynthesizing microorganisms

**Journal:** Applied Microbiology and Biotechnology

**Author's name:** Munehiko ASAYAMA

**Affiliations:** *Laboratory of Molecular Genetics, College of Agriculture, Ibaraki University, Ami, Inashiki, Ibaraki 300-0393, Japan*

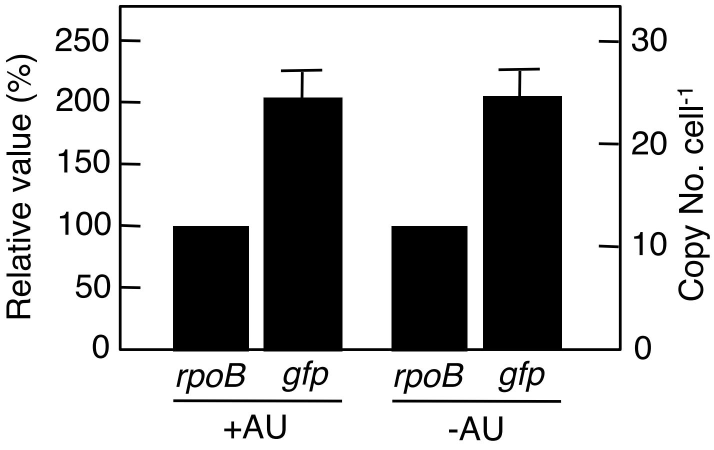

Relative value (%)
250
200
150
100
50
0
30
20
10
0
Copy No. cell-1
rpoB
gfp
rpoB
gfp
+AU
-AU

**Fig. S1** Q-PCR for analysis of copy number of expression vectors in the PCC 6803 transconjugants. Quantitative real-time PCR was done in a 30-μl reaction solution containing 1 μl of total DNA (prepared from the transconjugants: +AU = 6803_GFP500, -AU = 6803_GFP461c; see Table 1 of main manuscript), 20 pmol of the primers (6803rpoB-QF and 6803rpoB-QR for the *rpoB* gene in the genome, GFP-F and GFP-QRT for the *gfp* gene in the expression vector), and 15 μl of substrate mixture. The abundance of the PCR products was measured and presented as relative values (%) with standard deviation in triplicate experiments. Copy numbers are also shown and based on 100% of the value for the *rpoB* gene which exists as one copy in PCC 6803 genomic DNA (12 copies of genomic DNA per cell, see text of main manuscript).
